# Prospects of high-resolution resonant X-ray inelastic scattering studies on solid materials, liquids and gases at diffraction-limited storage rings

**DOI:** 10.1107/S1600577514017123

**Published:** 2014-08-31

**Authors:** Thorsten Schmitt, Frank M. F. de Groot, Jan-Erik Rubensson

**Affiliations:** a Research Department Synchrotron Radiation and Nanotechnology, Paul Scherrer Institut, Swiss Light Source, WSLA/123, 5232 Villigen PSI, Switzerland; b Inorganic Chemistry and Catalysis, Debye Institute for Nanomaterials Science, Utrecht University, Universiteitsweg 99, Utrecht 3584 CG, The Netherlands; c Department of Physics and Astronomy, Uppsala University, Box 516, Uppsala 751 20, Sweden

**Keywords:** X-ray emission, resonant inelastic X-ray scattering, X-ray absorption, diffraction-limited storage rings, molecules, correlated materials, transition metal ions

## Abstract

The spectroscopic technique of resonant inelastic X-ray scattering (RIXS) will particularly profit from immensely improved brilliance of diffraction-limited storage rings (DLSRs). In RIXS one measures the intensities of excitations as a function of energy and momentum transfer. DLSRs will allow for pushing the achievable energy resolution, signal intensity and the sampled spot size to new limits. With RIXS one nowadays probes a broad range of electronic systems reaching from simple molecules to complex materials displaying phenomena like peculiar magnetism, two-dimensional electron gases, superconductivity, photovoltaic energy conversion and heterogeneous catalysis. In this article the types of improved RIXS studies that will become possible with X-ray beams from DLSRs are envisioned.

## Abstract 2

Diffraction-limited storage rings will allow for pushing the achievable energy resolution, signal intensity and the sampled spot size in resonant inelastic X-ray scattering (RIXS) experiments to new limits. In this article the types of improved soft X-ray RIXS studies that will become possible with these instrumental improvements are envisioned.

## 1. Introduction   

In the spectroscopic technique of resonant inelastic X-ray scattering (RIXS) incident X-rays are inelastically scattered on atoms, molecules or solid materials, creating low-energy excitations of the electronic system. Such excitations can be measured as a function of momentum transfer **q** and as a function of the difference between the energies of the incident and scattered X-rays, *h*ν_out_ − *h*ν_in_. In a two-step picture of the RIXS process the system is first excited from the ground state to a core-excited intermediate state and then relaxes back to a low-energy excited final state (Ament *et al.*, 2011 ▶; Butorin, 2000 ▶; de Groot, 2001 ▶; Kotani & Shin, 2001 ▶). In addition to studying such low-energy excitations in valence-band RIXS one can also study core–core RIXS processes, for example to improve chemical sensitivity in comparison with X-ray absorption spectroscopy techniques. For both cases the involvement of a core-hole localizes the scattering event to a specific site in the system. Choosing the excitation energy differentiates between identical atomic species in different chemical environments. This enables RIXS to determine the oxidation state of atoms in a material. As dipole selection rules apply for both the photon-in and photon-out process, one can select particular orbitals and probe their symmetry with linearly polarized incident radiation. RIXS as a photon-in and photon-out spectroscopy is depth-sensitive and a powerful spectroscopic tool for the investigation of buried structures as well as liquids or gases that can be contained behind thin membranes. With the current state-of-the-art RIXS instruments one can probe vibrational (or phonon), charge, magnetic as well as orbital excitations with very high sensitivity with a resolving power of up to 12500 at 930 eV photon energy [Strocov *et al.*, 2010 ▶; Ghiringhelli *et al.*, 2006 ▶; see also the collection of papers on ‘Progress in Resonant Inelastic X-ray Scattering’ (Schmitt & Simon, 2013 ▶)]. Instrumental improvements of the RIXS technique are, at the time of writing, underway at several of the modern synchrotron radiation sources.

For small molecules, vibrational resolution will give access to fine details in the scattering dynamics and the involved states. One will be able to study consequences of the dipole selection rules and their limits in small symmetric systems. For larger molecules electronic vibronic coupling will be in the focus of research, and one vision is to use detuning to control the nuclear wavepacket in complex potential surfaces, which is relevant for the understanding of chemical reactions. For liquids and molecular materials the opportunity to map the local potential surfaces of the electronic ground state is a new unique tool. Analysing the natural line shapes associated with electronically excited states will give important information about the intermolecular interactions.

Coordination complexes, transition metal ions in solution and transition metals in proteins share the dominant effects of spin-orbit, dd- and charge transfer excitations with transition metal oxides (TMOs). Improved resolution of RIXS has the potential to reveal new details of the electronic structure. Increased signal intensity with the new instrumentation will enable probing RIXS involving so far unexplored weak electronic transitions.

For solid state materials one can study all elementary excitations within spin-, orbital-, charge- and lattice degrees of freedom of the solid. TMOs possess properties like ferro- and antiferromagnetism, metal–insulator transitions and superconductivity. Oxide heterostructures made of layered TMOs are constantly gaining in research interest as their special functionalities can be used for the design of device components in advanced technologies based on the electronic and magnetic materials properties. In the future, artificially designed electronic systems providing complex or coupled functionalities will be indispensable. Unconventional superconductors within TMOs and related material families are another important class of materials that is of high relevance for future improvement of energy generation, transmission and storage. Increased energy resolution in RIXS at diffraction-limited storage rings (DLSRs) will allow, for example, probing the relevant energy scale of the superconducting gaps. For all of these materials it will be useful to understand their low-energy properties at a sub-micrometre to nanometre length scale as electronic inhomogeneities and intrinsically heterogeneous materials need to be understood.

## 2. Instrumental opportunities of RIXS at DLSRs   

The spectroscopic technique of RIXS will particularly profit from the immensely improved brightness of DLSRs. The significantly reduced horizontal source sizes and horizontal beam divergence will allow the instrumental capabilities to be pushed even further in terms of achievable energy resolution, signal intensity and the sampled spot size. Refocusing optics schemes with large horizontal demagnification for long beamlines will make it possible to reach horizontal and vertical spot sizes at the sample position of sub-micrometre size. This will give RIXS the capability of sensitively analysing inhomogeneous samples as well as employing spectrometer concepts for parallel detection of incident and emitted photon energies in one single shot (Strocov, 2010 ▶; see also §4.2 and Fig. 4). Small horizontal source size and beam divergence will, furthermore, be beneficial when building long beamlines for extremely high energy resolution. In such beamlines the used optical surface of the mirrors along the beamline will be correspondingly decreasing with these beam parameters, which in turn will relax the limits set by the realistically attainable slope errors of the optical elements over large areas. All of this together will open totally new opportunities with more sophisticated RIXS experiments that will reveal increased information on the electronic properties of molecules and complex materials.

## 3. Molecules   

The high brilliance of ultimate storage rings can be used for increasing count rate and energy resolution in RIXS experiments on free molecules, liquids and molecular materials. Already at moderate energy resolution RIXS gives important information about local electronic structure and dynamics. Especially when investigating dilute samples (Lange *et al.*, 2012 ▶) and, for example, *in operando* catalytic processes (Kristiansen *et al.*, 2013 ▶), the method is particularly ‘photon hungry’. Therefore, improved photon flux in a tightly focused spot on the sample will directly improve the spectral quality.

**Figure 1:**
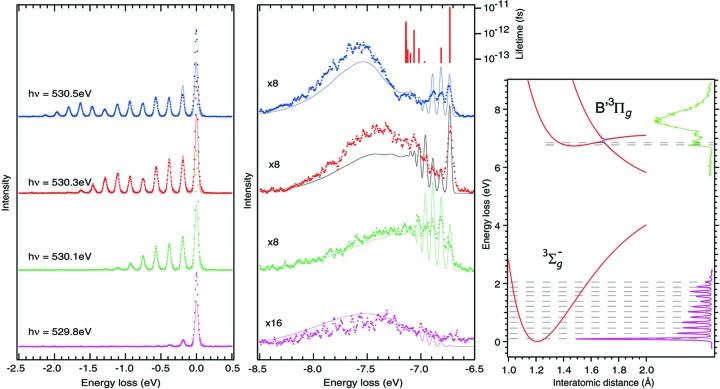
Excitation on the 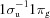 resonance in O_2_ populates vibrational excitations in the electronic ground state, and a complex structure associated with the avoided curved crossing of the first dipole-allowed electronically excited states. [From Hennies *et al.* (2010 ▶); Copyright (2010) by the American Physical Society.]

It is particularly rewarding to use the brilliance of ultimate storage rings for improved energy resolution. Only at the state-of-the-art beamlines of new generation synchrotrons has it in recent years become possible to resolve individual vibrations (Hennies *et al.*, 2010 ▶; Oura *et al.*, 2010 ▶; Rubensson *et al.*, 2012 ▶; Harada *et al.*, 2013 ▶) in RIXS spectra (see Fig. 1 ▶, left panel). This gives access to detailed information about the dynamics during the fast (femtosecond) scattering process. For fundamental molecular physics vibrationally resolved RIXS opens the door to a wealth of important interactions and phenomena, including vibronic electronic state interference, spin–orbit coupling dynamics, ultrafast symmetry breaking including core-hole localization, and wavepacket development at conical intersections.

**Figure 2:**
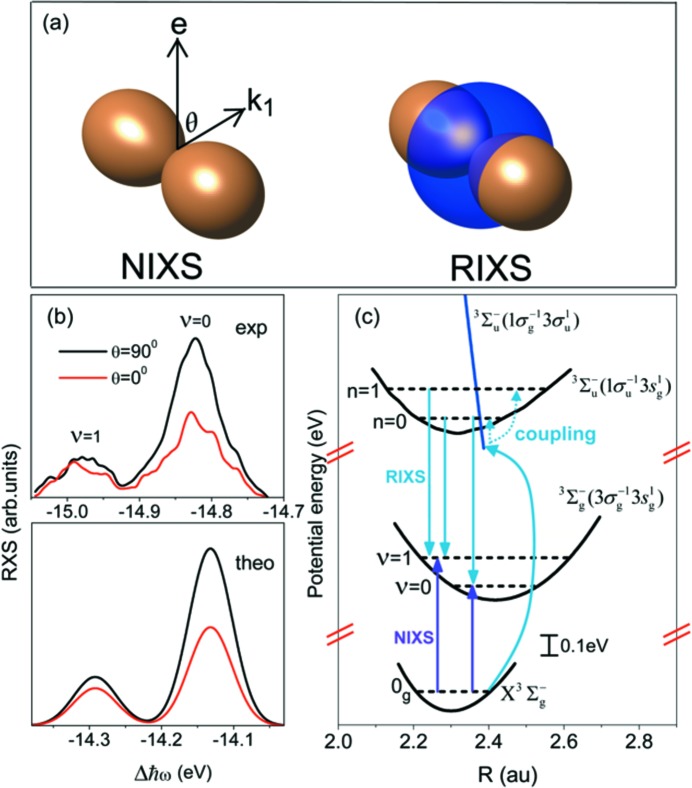
Excitation on the 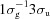 resonance in O_2_ reveals intriguing dynamics. First, coupling to the 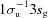 state is opening the channel to populate 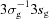 final states. Second, the angular anisotropy of the process demonstrates interference with the non-resonant inelastic scattering channel. [From Sun *et al.* (2013 ▶); Copyright (2013) by the American Physical Society.]

The O_2_ molecule has been used as a showcase, and several significant observations have been reported, comprising behaviour at avoided curve crossings (Hennies *et al.*, 2010 ▶) (see Fig. 1 ▶, middle panel) and spatial quantum beats (Pietzsch *et al.*, 2011 ▶) during dissociation. A new selection rule due to internal spin coupling has been established, and the breakdown of an accepted selection rule due to orbital parity has been observed (Sun *et al.*, 2011 ▶). Recently, an in-depth analysis showed that the non-resonant inelastic X-ray scattering channel must be taken into account to describe the polarization dependence of the vibrationally resolved spectra of O_2_ (see Fig. 2 ▶) (Sun *et al.*, 2013 ▶).

The investigation of these fundamental aspects of the scattering process in small molecules is important for further applications in the investigation of more complex systems of chemical and biological relevance. Whereas it is obvious that the experimental resolution will be the limiting factor in RIXS spectra of small molecules, where soft modes and possible *rotational* fine structure at sub-meV energies will continue to be out of reach, we emphasize in the following that high resolution will have an even larger impact in studies of complex systems.

Vibrationally resolved RIXS gives access to local ground-state potential surfaces in complex systems like large molecules, solutions and molecular materials. In principle the final states are the same as in IR Raman spectroscopy, and the development makes a comparison between the two methods relevant. The final states gain intensity in completely different ways in low-energy Raman and RIXS. While local modes in complex systems can often be isolated and identified in low-energy Raman, the RIXS process is intrinsically local due to the core hole in the intermediate state. As atomic species have different core-level energies, energy selectivity implies site selectivity, and it is obvious that oxygen *K* RIXS monitors the local structure at the oxygen site in a molecule like acetone (Sun *et al.*, 2011 ▶) and for dilute water in a solvent that does not contain oxygen atoms (Lange *et al.*, 2012 ▶). Whenever the chemical surrounding gives rise to specific features in the absorption signature, as, for example, –OH, –COO– and –C=O groups in molecular materials, different sites associated with the same species can also be separately investigated.

Whereas in IR Raman the first vibrational excitation and a few overtones are observed, a core excitation often initiates rather violent nuclear rearrangement, and therefore RIXS spectra map the local potential surface of the electronic ground state, with a large number of vibrational excitations sometimes all the way to dissociation (Pietzsch *et al.*, 2011 ▶), with the prospect of giving a unique picture of the local chemical bond in complex systems.

For fast fluctuating liquid systems it can be noted that the time scales for the two processes are different. While the duration of the RIXS process normally is a few femtoseconds, one period of IR radiation, and thus also IR scattering duration is much longer than RIXS. Thus, RIXS is the faster probe and the averaging over various conformations in a fluctuating liquid is different in the two methods.

In small molecules the separation between vibrational sub-levels is typically of the order of 100 meV, which can be readily resolved already today. In molecular materials local vibrational modes sometimes have similar energies, for example, in inorganic and coordination compounds (Nakamoto, 2006 ▶), although also softer modes, reaching down to *kT* at room temperature (25 meV) and below are observed in IR Raman spectra, and must also be present in RIXS spectra with appropriate resolution. Vibrationally resolved RIXS spectra of liquids (Harada *et al.*, 2013 ▶; Sun *et al.*, 2011 ▶; Rubensson *et al.*, 2013 ▶) have a large potential in systems relevant for chemical and biological applications. We note that, for example, the C=O stretch vibrations of peptide linkages of the amide I band in IR spectra are sensitive to structural properties and the hydrogen bond environment (Zanetti-Polzi *et al.*, 2013 ▶). This band is around 12–13 meV wide and experiences shifts of the order of a few meV. In condensed water, librational modes are of the order of 50–60 meV, the hydrogen bond stretch modes lead to features at around 6 meV and 23 meV in the IR spectrum, and small shifts of these features depend on phase, temperature and substitution. Phonon excitations in liquid water, readily observed in non-resonant inelastic X-ray scattering (Sampoli *et al.*, 1997 ▶), will require an energy resolution on the order of 1 meV.

To exploit the full power of RIXS on molecular systems it is therefore worthwhile to improve the energy resolution further. At ultimate storage rings we believe it will become possible to measure RIXS spectra with the required resolving power, around 50000, at the O *K* edge (around 10 meV), which will be sufficient for the investigation of a plethora of phenomena which are today unreachable.

Experimentally, this implies that new demands have to be met. As soon as soft modes are involved one expects that RIXS spectra are strongly temperature dependent, and it was indeed found that a finite temperature must be assumed to describe oxygen *K* RIXS spectra of liquid acetone, measured at room temperature. As the excitations of the order of *kT* can be easily resolved, temperature control will be crucial in general. We note also that determination of small energy shifts with high precision put new requirements on the instruments. Although calibration of the energy dispersion can be performed with high accuracy, for example using well known vibrational energies of isolated diatomic molecules (Hennies *et al.*, 2010 ▶), such experiments put strong demands on stability.

In electron spectroscopy the removal of an electron induces a screening relaxation in the chemical surrounding with a rather large influence in the spectra. This has no counterpart in vibrational RIXS, but for electronically excited final states intermolecular dynamics does have a subtle influence on the line shapes. This is the case, for example, when an electronic excitation is associated with a change in local dipole moment. In a weakly interacting liquid like acetone it was found that the broadening due to intermolecular dynamics in the O *K* RIXS spectra is much smaller than the broadening due to intramolecular soft modes, but larger effects are predicted for acetone in aqueous solution (Sun *et al.*, 2011 ▶). We believe that the natural line shape in RIXS spectra of condensed molecular systems will in general be a sensitive probe of intermolecular dynamic interactions, for example, associated with a ‘sudden’ change in local dipole moment. Although the line-shape analysis requires some theory development, we believe that the inherent broadening often is so large that the demands on energy resolution is more relaxed than the requirements for vibrations in the electronic ground state. Indeed, the analysis of the acetone spectra seems to indicate that the natural line shapes are measured, without much experimental contribution to the width, already at around 50 meV resolution.

The dipole approximation is frequently used, and it seems to be applicable to most molecular RIXS phenomena in the sub-keV region. In particular, the parity selection rule in inversion symmetric systems seems to be valid within today’s experimental accuracy, and *via* control of the polarization state of the incoming radiation the angular anisotropy of the emitted radiation due to π/σ symmetry of the involved states seems to follow the dipole approximation. Note, however, that the condition for strict validity of the dipole approximation, 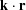 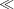 1, is hardly fulfilled over the whole soft X-ray range. In addition, non-dipole effects at rather low energies have been observed in horizontal-plane angular anisotropies in electron emission spectra (Guillemin *et al.*, 2005 ▶). At many of the upcoming RIXS set-ups there will be the opportunity to investigate angular dependence of molecular RIXS in the horizontal plane. As with all new opportunities it will be investigated in exploratory fashion for small model systems, with the prospect for application in more complex systems.

An experimental challenge at ultimate storage rings is to perform molecular RIXS experiments using Laguerre–Gauss (LG) beams with well defined orbital angular momentum (OAM). Such an experiment requires a highly coherent beam, and a tight focus, of the order of 0.1 µm. It has been predicted (Sasaki & McNulty, 2008 ▶) and recently also demonstrated (Bahrdt *et al.*, 2013 ▶) that undulators emit radiation with well defined OAM already at third-generation synchrotrons, and we anticipate that undulators at the new DLSRs will emit intense LG beams. Such beams have had a large impact in optical physics, and it has been predicted that the OAM of the radiation will have a large impact on RIXS spectra of molecules (Rury, 2013 ▶). The coupling between the LG field and the nuclear motion of the molecule represents an electromagnetically induced vibronic coupling of the molecular degrees of freedom. The long-term prospect of introducing OAM radiation in RIXS spectroscopy is the control of the vibronic electronic coupling in complex systems and processes. For the initial experiments we propose to put the predictions of Rury (2013 ▶) on naphthalene and other aromatic molecules to an experimental test.

## 4. Correlated electron materials   

### 4.1. High-temperature superconductors   

One of the most important questions within the physics of correlated electron materials addresses the pairing mechanism in high-temperature superconductors. Spin fluctuations are currently regarded as one of the prominent candidates for delivering the superconducting glue in unconventional superconductors. It has recently been theoretically understood that transition metal *L*-edge RIXS (2*p*–3*d* RIXS) can probe single spin excitations due to the strong spin–orbit coupling of the 2*p* core hole (Ament *et al.*, 2009 ▶; Haverkort, 2010 ▶). Together with the large improvement of the energy resolution of the RIXS technique with soft X-rays, this motivated many studies of the high-energy spin excitations in cuprate and iron pnictide superconductors and their parent compounds (Braicovich *et al.*, 2010 ▶; Guarise *et al.*, 2010 ▶; Le Tacon *et al.*, 2011 ▶; Dean *et al.*, 2013*a* ▶,*b* ▶; Zhou *et al.*, 2013 ▶). For two-dimensional long-range antiferromagnetically ordered cuprates the momentum dependence of single- and multi-magnons can readily be probed with Cu *L*_3_ RIXS as first demonstrated for La_2_CuO_4_ and Sr_2_CuO_2_Cl_2_ (Braicovich *et al.*, 2010 ▶; Guarise *et al.*, 2010 ▶). For the hole-doped cuprates these spin excitations persist as paramagnon-excitations that are only marginally softening as a function of doping and are broadened due to the interaction with electron–hole pair excitations for underdoped, optimally doped as well as overdoped cases (Le Tacon *et al.*, 2011 ▶; Dean *et al.*, 2013*a* ▶.*b* ▶). For electron-doped Nd_2–*x*_Ce_*x*_CuO_4_ the magnon dispersion is in contrast hardening as a function of Ce doping (Lee *et al.*, 2013*a* ▶; Ishii *et al.*, 2014 ▶). Recent theoretical treatment has challenged the magnetic origin of these paramagnon-excitations and could describe those in a Fermi liquid-like model of itinerant electrons based solely on the underlying band structure (Benjamin *et al.*, 2014 ▶).

For parent BaFe_2_As_2_ and optimally hole doped Ba_1–*x*_K_*x*_Fe_2_As_2_ iron-pnictides it has been experimentally demonstrated that Fe *L*_3_ RIXS is sensitive to collective spin excitations also for this electronically more delocalized unconventional superconductor (Zhou *et al.*, 2013 ▶). From these Fe *L*_3_ RIXS measurements it was found that the high-energy spin excitations persist also in the superconducting phase of iron pnictides, whereby the spin dispersion curve of optimally hole doped Ba_1–*x*_K_*x*_Fe_2_As_2_ is clearly softening relative to undoped BaFe_2_As_2_ (Zhou *et al.*, 2013 ▶).

Improved energy resolution for RIXS at future DLSRs will enable the study of the spin excitations in materials with smaller super-exchange interactions between 10 meV and 100 meV. This will allow studying the spin dynamics in, for example, iron selenides, nickelates, cobaltates and manganites. For unconventional superconductors it will be interesting to increase the energy resolution below the energy scale of the superconducting gaps. It has been suggested by theory that the dynamical structure factors of charge and spin that can be probed with momentum-dependent RIXS can give direct access to the pairing symmetry and the phase of the order parameter (Marra *et al.*, 2012 ▶). In order to be able to realise this possibility one will need to measure the spin excitations with the highest resolution possible around the middle of the Brillouin zone (0, 0) and as close as possible towards (0.5, 0.5). A recent exact diagonalization and quantum Monte Carlo study confirmed the importance of low-energy paramagnons for the pairing interaction (Jia *et al.*, 2014 ▶). The low-energy spin excitations in this theory investigation show a clear dependence on doping and the emergence of ferromagnetic correlations that can only be probed with Cu *L*_3_ RIXS experiments with considerably improved resolution. Analysing the polarization conditions of the scattered beam in RIXS will allow the character of the detected excitations to be assessed (Ghiringhelli & Braicovich, 2013 ▶). Such analysis will make it possible to discriminate between spectral contributions from charge and spin excitations.

### 4.2. Quasi one-dimensional cuprates   

**Figure 3:**
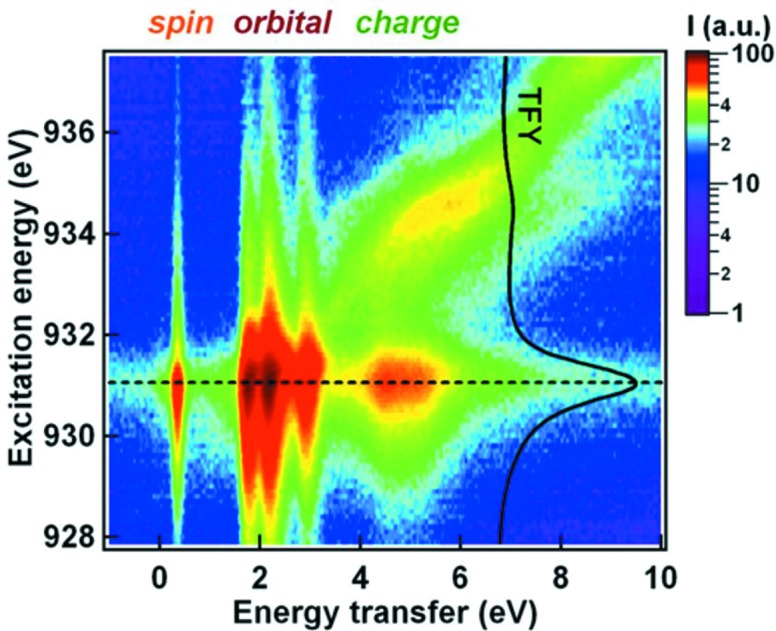
Cu *L*_3_ RIXS map of Sr_2_CuO_3_ displaying the incident excitation energy dependence of spin, orbital- and charge excitations. [From Schlappa *et al.* (2012 ▶).]

**Figure 4:**
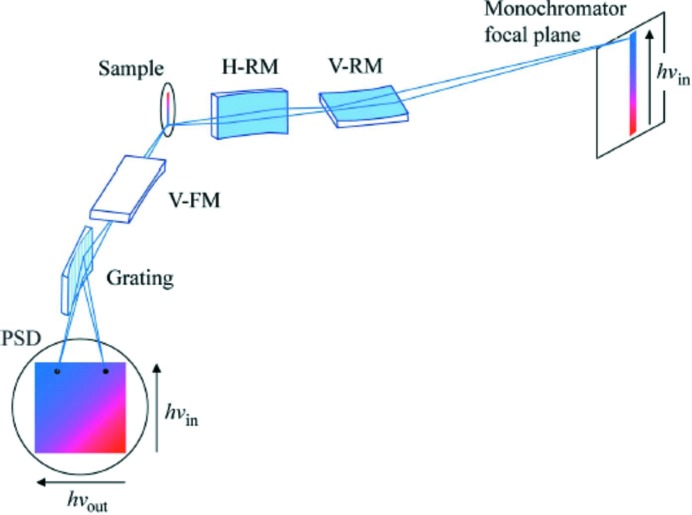
Principle optical layout of a RIXS spectrometer for parallel acquisition of RIXS intensity maps *versus* incident and emitted X-ray energies. [From Strocov (2010 ▶).]

Several fundamental scientific problems relevant for the physics of one-dimensional materials have been addressed within RIXS studies on quasi one-dimensional cuprates made out of connected CuO_4_ plaquettes. In particular, a study on the corner-sharing single-chain compound Sr_2_CuO_3_, possessing the nearly ideal properties of a one-dimensional Heisenberg spin-1/2 system, demonstrated the strength of RIXS in studying spin and orbital interactions. The RIXS intensity map of Sr_2_CuO_3_ consisting of many single RIXS spectra, depicted in Fig. 3 ▶ as a function of incident photon excitation energy across the Cu *L*_3_ resonance and energy transfer of the inelastically scattered X-rays, reveals the elementary excitations within spin, orbital and charge degrees of freedom as well as the emergence of fluorescence for incident energies higher than the main resonance. Such RIXS maps will be able to be acquired in parallel simultaneous detection of incident and detected photon energies with new spectrometer concepts at DLSRs that use a vertical line focus of the incident beam on the sample combining vertical imaging and horizontal dispersion in the spectrometer optics (Strocov, 2010 ▶). A suggestion by V. N. Strocov for such an imaging type of RIXS spectrometer set-up is shown in Fig. 4 ▶. For electronic systems with partially occupied *d*-shell and more complicated electronic ground states than the relatively simple *d* ^9^ valence configurations of insulating cuprates, such RIXS maps will be particularly powerful in obtaining a quick overview about existing electronic excitations and their resonating behaviour.

**Figure 5:**
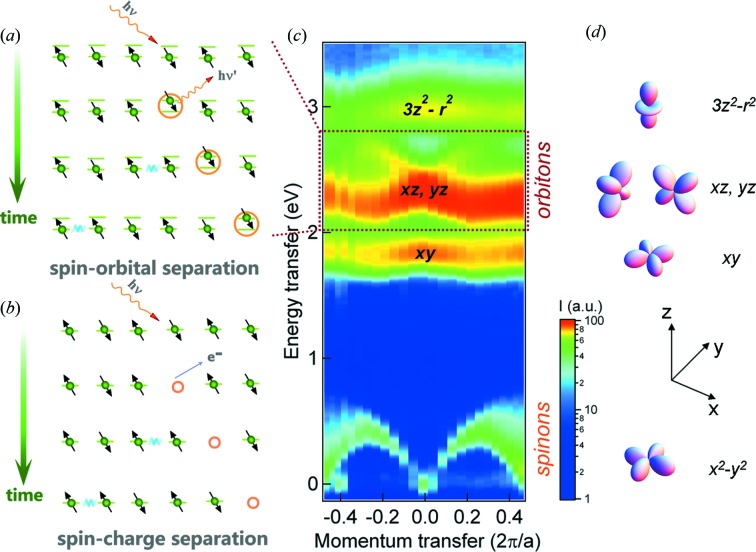
Spin-orbital (*a*) and spin-charge (*b*) separation spectroscopic processes for a single spin chain with RIXS and ARPES, respectively. (*c*) RIXS intensity map of the dispersing two-spinon (and higher-order spinons) and orbiton excitations *versus* photon momentum transfer along the chains and photon energy transfer. (*d*) Illustration of the involved Cu 3*d* orbitals [Reproduced and adapted from Schlappa *et al.* (2012 ▶); and Schmitt *et al.* (2013 ▶), *J. Electron Spectrosc. Relat. Phenom.* **188**, 38–46, Copyright (2013), with permission from Elsevier.]

The momentum transfer dispersive Cu *L*_3_ measurements of Sr_2_CuO_3_ depicted in Fig. 5(*c*) ▶ reveal magnetic two-spinon and collective orbital excitations in the energy loss ranges 0–700 meV and 1.5–3.2 eV, respectively (Schlappa *et al.*, 2012 ▶). Theoretical analysis of the data showed that the orbital excitations break up in spinons and orbitons, the quasi-particles of the spin and orbital degree of freedom, respectively [see illustration of the process in Fig. 5(*a*) ▶]. The developed model employing super-exchange processes (interaction between spin- and orbital degrees-of-freedom) as well as a novel spin-orbital separation phenomenon allows the experimental data to be simulated very well (Schlappa *et al.*, 2012 ▶) [see Fig. 5(*a*) ▶ for an explanation of the involved spectroscopic processes]. This allowed the first unambiguous experimental observation of collective orbital excitations.

The two-leg spin ladder consisting of two parallel chains (legs) with transverse (rung) exchange coupling is a step between ideal one-dimensional and two-dimensional behaviour (Dagotto & Rice, 1996 ▶). The magnetic ground-state is formed from spin-singlets, for which the magnetically excited state can be reached by singlet-to-triplet excitations, so-called triplons. Cu *L*_3_ RIXS on the quantum spin-liquid cuprate material Sr_14_Cu_24_O_41_, consisting of a two-leg spin ladder and an edge-sharing spin chain, could probe the momentum dispersive two-triplon magnetic excitations along the leg direction of the two-leg ladder (Schlappa *et al.*, 2009 ▶). Higher spectral resolution of the order of 10 meV will be needed for such spin ladder systems in order to analyse in detail the magnetic gap as well as to disentangle spectral contributions from intra-ladder and in-chain magnetic exchange coupling and from optical phonons.

Li_2_CuO_2_ and CuGeO_3_ are prototype edge-sharing chain compounds with weak super-exchange coupling between Cu spins due to a Cu—O—Cu bond angles close to 90°. Below *T*_N_ = 9 K antiferromagnetic and ferromagnetic correlations are dominating in Li_2_CuO_2_ between and within the chains, respectively. For CuGeO_3_ short-range antiferromagnetism coexists with frustrated magnetic interactions driving this system for low temperatures (*T*_SP_ = 14 K) through strong fluctuations into a spin-Peierls phase. O *K*-edge RIXS spectra of Li_2_CuO_2_ and CuGeO_3_ could clearly resolve strong charge transfer related spectral components around 3.5 eV energy loss (Monney *et al.*, 2013 ▶). For CuGeO_3_ these non-local charge transfer excitons are increasing in intensity when cooling towards *T*_SP_. In contrast to this, temperature-dependent measurements for Li_2_CuO_2_ show evidence of a clear opposite temperature behaviour for the corresponding spectral component. From pd-Hamiltonian cluster calculations this exciton has been identified as a Zhang–Rice singlet exciton on neighbouring CuO_4_ plaquettes, enabling thereby highly sensitive probing of the temperature-dependent short-range spin-correlations in edge-shared cuprate spin chains (Monney *et al.*, 2013 ▶).

The electron-lattice coupling can be determined from O *K*-edge RIXS as demonstrated in edge-sharing spin-chain cuprates (Lee *et al.*, 2013*b* ▶). For Ca_2+5*x*_Y_2–5*x*_Cu_5_O_10_ a 70 meV lattice vibrational mode and several higher orders of this mode could be resolved (Lee *et al.*, 2013*b* ▶). The vibrational progression of such measurements can thereby provide a direct measurement of the electron–lattice coupling strength. We expect that increased resolution and spectral quality at DLSRs will establish RIXS as a method for measuring the electron–lattice coupling also in systems of smaller optical phonon energy scale.

### 4.3. Correlated transition metal oxides   

Spin and orbital degrees of freedom of strongly correlated transition metal oxides are strongly coupled through their super-exchange interactions. The balance of these interactions with the crystal field of the lattice degree of freedom gives, for example, rise to orbital quantum fluctuations and the occurrence of orbital order. For cases with dominating super-exchange interaction, it is expected that their orbital excitations are of a collective nature and show sizable dispersion as orbital waves termed orbitons. Soft X-ray *L*-edge RIXS has recently been applied to the orbitally degenerate Mott insulators YTiO_3_, LaTiO_3_ (Ulrich *et al.*, 2009 ▶) and YVO_3_ (Benckiser *et al.*, 2013 ▶), that revealed evidence for the dominance of two-orbiton scattering, rather speaking for a situation where both crystal field effects and super-exchange need to be considered on equal footing (Ulrich *et al.*, 2009 ▶; Benckiser *et al.*, 2013 ▶). Significantly higher spectral resolution of better than 10 meV achievable with RIXS at DLSRs will allow better discrimination of both contributions and unambiguous observation of single orbitons, if they exist in the respective systems studied.

The competition of the interactions in all degrees of freedom in transition metal oxides gives rise to phase transitions like, for example, the intriguing metal–insulator transitions (MITs) characterized by dramatic changes in the resistivity (Imada *et al.*, 1998 ▶). Many MITs are driven by electron correlation effects referred to as Mott transitions (Imada *et al.*, 1998 ▶). Crystallographic distortions and related electron–phonon interactions can also be coupled to the MIT, in which case the transition is categorized as a Peierls transition (Imada *et al.*, 1998 ▶). For the prototypical examples of such MIT materials as VO_2_ and V_2_O_3_, it has been recognized by infrared microscopy and scanning photoemission microscopy that metallic domains of sub-micrometre size already show up at the onset of the temperature-dependent MIT (Qazilbash *et al.*, 2007 ▶; Lupi *et al.*, 2010 ▶). Spatially resolved scanning RIXS experiments in the 10–100 nm range that will become feasible employing extreme refocusing optics schemes or Fresnel zone plate optics at DLSRs will help to understand the origin of such electronic inhomogeneities. This will, furthermore, be useful for gaining in general a better understanding of electronic excitations within all degrees of freedom in materials showing already intrinsic phase separation.

### 4.4. Thin films of oxide heterostructures   

With thin films of oxide heterostructures one can achieve special functionality for devices like high-electron mobility transistors or magnetic tunnel junctions. TMOs with perovskite structure are especially suitable for creating such heterostructures since they display competing ordering phenomena like ferromagnetism, antiferromagnetism, metal–insulator transitions, superconductivity or multiferroic behaviour (Mannhart & Schlom, 2010 ▶; Takagi & Hwang, 2010 ▶). These materials properties are caused by the interplay of orbital, lattice, charge and spin degrees of freedom (Dagotto, 2005 ▶). Understanding the details of the physical phenomena that can be connected to these degrees of freedom is pre­requisite for engineering interactions in future devices.

The bulk probing capability of RIXS makes it extremely powerful for analysing single material layers or the interface between different layers of oxide heterostructures. RIXS at the Ti *L*_3_-edge revealed different spectral signatures of the localized and delocalized Ti 3*d* carriers at the two-dimensional conductive interface between the band insulators LaAlO_3_ and SrTiO_3_ (Zhou *et al.*, 2011 ▶; Berner *et al.*, 2010 ▶). Furthermore, such investigations allow quantifying the energy separation between the respective crystal field levels, which provides information on local structural distortions. The sensitivity of magnetic RIXS at the Cu *L*_3_-edge in fundamental physics questions connected to oxide heterostructures has recently been illustrated for the problem of the spin excitation response in confined one and two unit-cell thick layers of La_2_CuO_4_ (Dean *et al.*, 2012 ▶). The investigated samples for this study consisted of La_2_CuO_4_/LaAlO_3_ multilayers, where LaAlO_3_ was used as the buffer material, to create ideal two-dimensional cuprate layers. It was unexpectedly found that the dynamic magnetic properties of such thin isolated layers differ by only a small degree from those of thick bulk samples, and were not showing any evidence for resonating valence bond correlations (Dean *et al.*, 2012 ▶). RIXS at O *K*-edges in a study of superconducting [(Ba_0.9_Nd_0.10_)CuO_2+δ_]_2_/[CaCuO_2_]_2_ superlattices could provide sensitive information on the occupied O 2*p* density of states of the insulating layer components. Observation of Zhang–Rice singlet excitons in such high-temperature superconducting heterostructures can, in addition, provide vital information on the charge transport between the charge reservoir and the superconducting infinite layer (Freelon *et al.*, 2006 ▶). Monitoring magnetic excitations and the ligand field properties of interfaces in (CaCuO_2_)_*n*_/(SrTiO_3_)_*n*_ superlattices with RIXS allows revealing the interfacial reconstruction of the Cu—O coordination and the reduction of the in-plane super-exchange for decreasing number of CaCuO_2_ layers (Minola *et al.*, 2012 ▶). Measurements of CaCuO_2_ thin films of different thickness on substrates of opposite epitaxial strain effect revealed, furthermore, how magnetic interactions and orbital energy scales can be tuned by variations of the lattice constants (Minola *et al.*, 2013 ▶).

RIXS studies on thin films of oxide heterostructures will naturally profit from the increased energy resolution at DLSRs as a solid understanding of the excitations in all degrees of freedom is needed for assessing the potential functionality of such heterostructures. Scanning submicrometre RIXS will allow, furthermore, evaluating to what degree electronic inhomogeneities limit applicability and performance of model devices for specific functionalities.

## 5. Transition metal ions in solids, coordination compounds and proteins   

The improved resolution and intensity of DLSR sources will also provide a number of new spectroscopic tools such as ultra-sharp X-ray absorption spectra of transition metal ions in essentially any system, including solid oxides, coordination compounds and proteins.

### 5.1. Ultra-sharp X-ray absorption spectra   

In hard X-ray absorption experiments, the effective spectral broadening can be drastically reduced with the so-called high-energy-resolution fluorescence detection (HERFD) technique (Hämäläinen *et al.*, 1991 ▶; de Groot *et al.*, 2002 ▶). In principle, such an approach is also possible in the soft X-ray range when recording complete RIXS maps (see also §4.2). The *L*_2,3_ edges of 3*d* transition metal systems are typically broadened with a 400 meV (FWHM) Lorentzian broadening due to the lifetime of the 2*p* core excited state. If the final state of the RIXS experiment will have a longer lifetime, in other words a sharper spectrum, it is also possible to measure the *L* edges with this improved resolution, provided that the experimental resolution is also similar to this broadening. For example, a dd-excitation final state can be used to scan through an *L* edge and such an approach should yield a spectral shape with an improved effective lifetime broadening, where it should be noted that the observed spectral shape will not necessarily be exactly equal to the X-ray absorption spectral shape due to state-dependent decay processes (de Groot *et al.*, 1994 ▶; Kurian *et al.*, 2012 ▶). Actually such state-dependent decay processes can teach us new details regarding the electronic structure, if they are better understood and simulated theoretically. It would be great to observe an *L*-edge (like) spectral shape with a 10 meV resolution instead of 400 meV resolution. Much new details will appear with such improvement in effective resolution which will force a more detailed spectral simulation and as such new insights into the local electronic structure of transition metal ions.

### 5.2. *L*_1_ X-ray absorption edges   

The *L*_1_ edge related to the excitation of the 2*s* core state is not popular mainly due to its large lifetime broadening that blurs any fine structure. However, in 2*s*2*p* (or 2*s*3*p*) RIXS it is possible to replace the 2*s* lifetime broadening by the 2*p* (3*p*) lifetime broadening. If it is possible to measure high-resolution 2*s* X-ray absorption spectra, these spectra are likely to be superior in resolution over the 1*s* X-ray absorption spectra (de Groot *et al.*, 2009 ▶). This gives them the potential to determine accurate electronic structure information, for example on the 2*s* to 3*d* quadrupole pre-edges in relation to the 2*s* to 4*p* edge excitations and in particular their coupling. The comparison of such high-resolution *L*_1_ edges with high-resolution *K* edges will reveal new details in the electronic structure of transition metal systems.

### 5.3. Resonant X-ray Raman experiments   

An intrinsically weak X-ray scattering process is the so-called resonant X-ray Raman process, also known as high-energy resolution off-resonance spectroscopy (HEROS). In the HEROS technique one excites at an energy before an absorption edge and detects a core–core X-ray emission channel, for example exciting the *K* edge of Ni metal 20 eV before its first structure and detecting the 1*s*2*p* X-ray emission spectrum. Recently, Szlachetko and co-workers have further advanced the use of HEROS in the hard X-ray range (Szlachetko *et al.*, 2013 ▶). They showed that the 1*s*2*p* X-ray emission spectrum is equivalent to the *K*-edge X-ray absorption spectrum, where the lifetime broadening of the 1*s* core hole is (approximately) replaced by that of the 2*p* core hole. The HEROS spectrum is equivalent to the high-energy-resolution fluorescence detected (HERFD) spectrum, because the 1*s* core state is only reached virtually and the sharpness of the 2*p* final state determines the broadening. A practical limitation of HEROS experiments is that one needs an X-ray emission channel with lower emission energy than the X-ray absorption edge, in other words as a detector channel a core–core X-ray emission must be used. This implies for the 2*p* core states of the 3*d* transition metals that only the 2*p*3*s* X-ray emission can be used. A crucial experimental aspect is that the excitation is before an edge, implying a constant and relatively deep X-ray penetration, also with the consequence of measuring a bulk spectrum without saturation effects.

## 6. Rare earth and actinide systems   

The 3*d* and 4*d* XAS spectra of rare earths are dominated by strong multiplet effects. The 3*d*4*f* and 4*d*4*f* RIXS spectra allow the determination of the low-energy excitations in rare earth systems. One interesting application is the determination of the crystal field effects on the 4*f*4*f* excitations. The crystal field effects in rare earth systems are typically of the order of 30 meV, which potentially will be directly detectable with the achievable resolution. However, as has been shown by Butorin, it is possible to obtain insights into the 4*f* crystal field effects also from the admixture of atomically forbidden transitions (Butorin, 2000 ▶).

**Figure 6:**
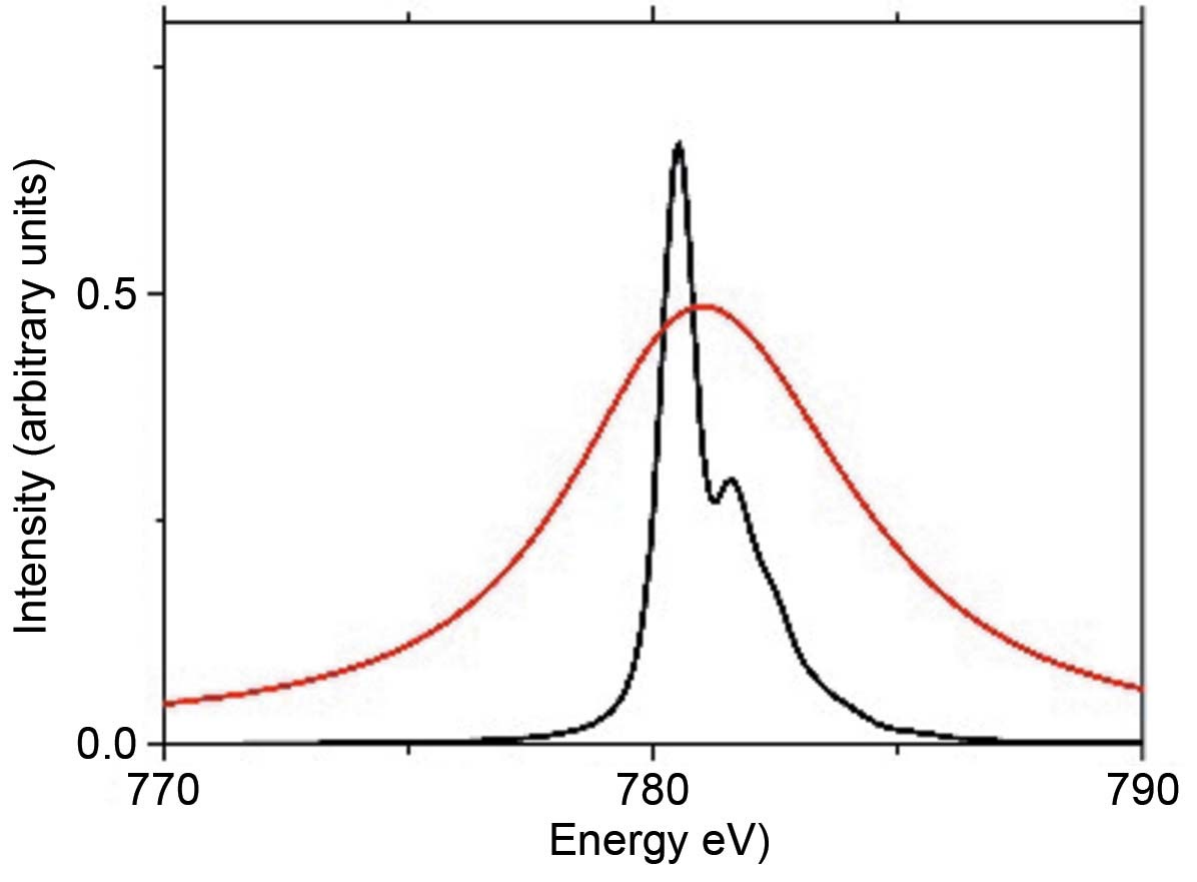
The calculated *N*_4_ edge of U^4+^ shows a single Lorentzian with 6 eV lifetime broadening (red). With 0.5 eV lifetime broadening, fine structure becomes visible (black line). Reprinted from Kvashnina & de Groot (2014 ▶). *J. Electron Spectrosc. Relat. Phenom.* **194**, 88–93, Copyright (2014), with permission from Elsevier.]

The actinides can be studied with their 4*d* and 5*d* XAS spectra. One important tool available is the usage of the RIXS detection to remove the lifetime broadening of the 4*d* core state. Fig. 6 ▶ shows the difference between a normal 4*d* XAS spectrum of U^4+^ and a RIXS detected 4*d* XAS spectrum. The RIXS detected spectrum is, as discussed in §5.1, much sharper and new spectral details become visible (Kvashnina & de Groot, 2014 ▶).

### 6.1. Interference effects in solids   

**Figure 7:**
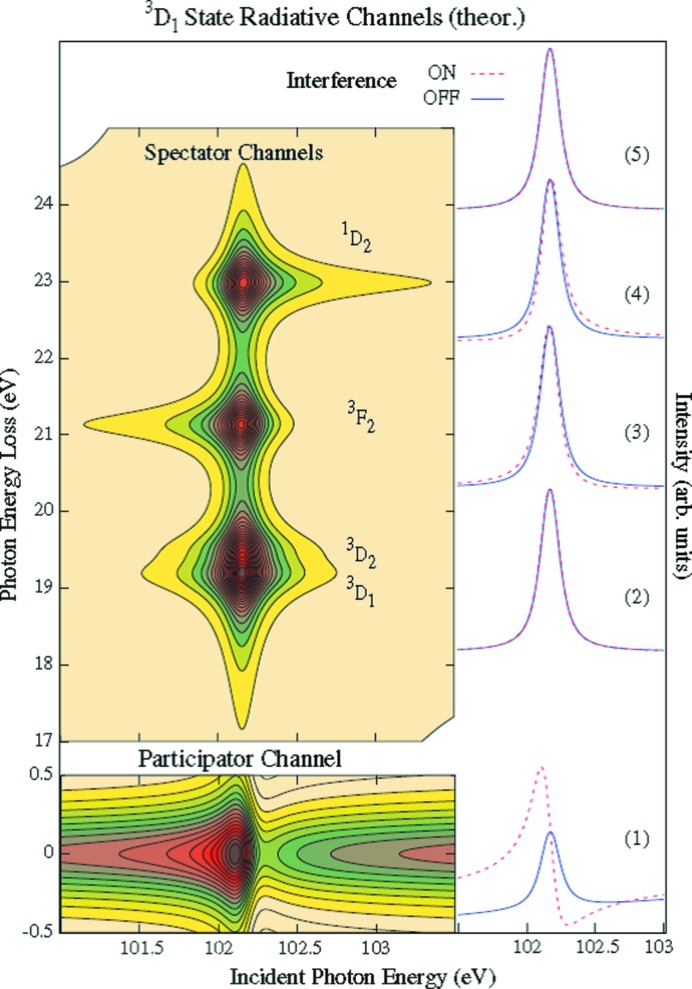
Model of resonant inelastic X-ray scattering across the ^3^ *D*_1_ La *N*-edge resonance, taking symmetry entanglement and channel interference into account. Right panel: partial fluorescence yield (1–4) resulting from integrating the RIXS energy scale (hatched line). (5) is the sum of (3)–(4). Neglecting channel interference leads to Lorentzian line profiles (full line). [From Suljoti *et al.* (2009 ▶); Copyright (2009) by The American Physical Society.]

It has been shown by Suljoti *et al.* (2009 ▶) that excitations at the 4*d* pre-edges interfere with the Lorentzian tail of the white line, giving rise to interference effects. Fig. 7 ▶ shows the energy dependence of the 4*d*5*p* RIXS plane, as the tails towards positive and negative energy are clearly visible. In principle it is possible to transfer these interference effects into the time domain to study the timescales of the various decay channels. The improved resolution of DLSR sources might allow the study of interference effects between states in transition metal systems, for example the difference between dd-excitations and charge transfer excitations, the differences between different valence and spin states, *etc*.

## 7. Active materials   

Active materials, including heterogeneous catalysts, fuel cells, hydrogen storage, batteries and light-capture materials, can be studied with RIXS under working conditions. Because RIXS is a photon-in photon-out probe it is possible to design reactor systems that allow the study of materials in action. Using the element selectivity of RIXS one can neglect the effects of the reactor itself apart from a partial attenuation of the X-ray beam.

### 7.1. Making the active sites visible   

The determination of the nature of the active site in a background of silent states is important in the study of active materials, for example catalyst systems. The information on the active site is difficult to obtain as it often concerns a minority of all states of a certain element. In standard X-ray techniques therefore, any signal originating from active states is overlaid by the signal from the inactive states.

**Figure 8:**
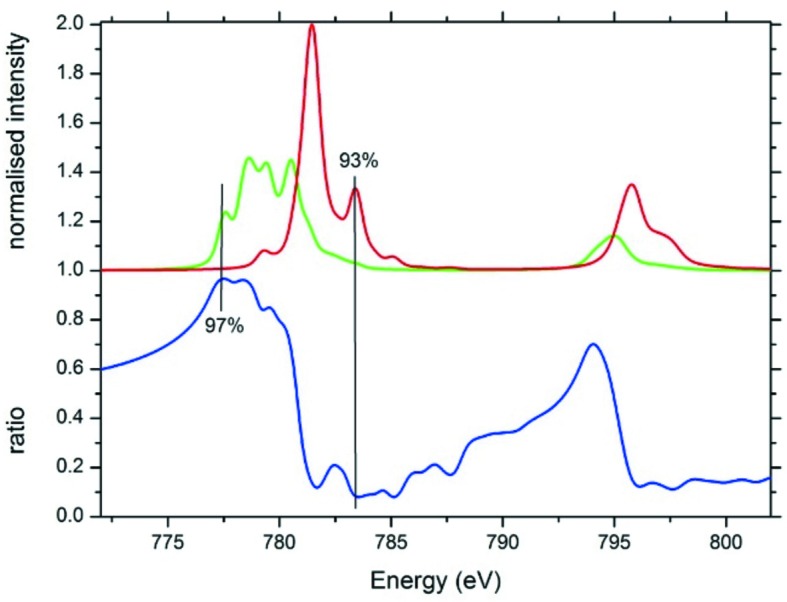
Theoretical 2*p* XAS spectra of typical Co^2+^ (green) and Co^3+^ (red) oxides. The blue spectrum indicates the fraction of the Co^2+^ spectral intensity calculated as [Co^2+^]/([Co^2+^] + [Co^3+^]).

Fig. 8 ▶ shows the simulated Co 2*p* XAS spectra of the octahedral Co^2+^ and Co^3+^ sites of Co_3_O_4_. The bottom curve shows the ratio of the two spectra, calculated as the fraction of the Co^2+^ spectral shape. One finds that at 775.5 eV the Co^2+^ spectrum dominates with 97% of the intensity. The detection of the RIXS spectrum at this energy will effectively only show the dd-excitations of Co^2+^, allowing a determination of its electronic structure. This procedure allows the determination of just a few % of a specific valence in a background of other valences. Similar procedures could be designed for other properties such as spin and local geometry.

### 7.2. Spatial resolution   

Over the last years the spatial resolution of X-ray absorption has been improved to 10 nm. A number of transmission X-ray microscopy (TXM) beamlines operate worldwide and the experiments involve *ex situ* vacuum experiments, but also experiments on *in vivo* biological systems and *in situ* active chemical systems. For example, de Smit and co-workers studied an iron-based Fischer–Tropsch catalyst in which both the iron *L*-edge spectra of the catalyst and the carbon *K*-edge spectra were analysed with 30 nm spatial resolution, allowing a correlated view on both catalyst and product formation (de Smit *et al.*, 2008 ▶). In comparison with X-ray absorption, RIXS is much richer in chemical accuracy, including the selective detection of certain states. A spatial resolution of, say, 100 nm will allow the accurate determination of such minority states in a material.

## 8. Summary and conclusions   

The main improvements for RIXS at DLSR sources are (*a*) increased signal intensity, (*b*) smaller sampled spot sizes and (*c*) increased energy resolution. In addition it can also be expected that coherent beams with well defined OAM will trigger developments that can be used to control or dis­entangle vibronic electronic coupling in materials.

### 8.1. Improved signal intensity   

The increased signal intensity allows experiments that currently suffer from low intensity, for example diluted systems or systems inside reactors under active conditions. One can design more robust high-temperature high-pressure reactors for experiments in the liquid state or under a gas atmosphere and use the increased signal intensity to detect a measurable signal under such demanding conditions. For example, the detection of the element and valence selective dd- and charge transfer excitations of a working catalyst system will require much higher signal strength than achievable today at third-generation synchrotron sources. Another range of experiments that will become much more feasible are RIXS experiments on metal centres in proteins using liquid jet sample environments.

### 8.2. Improved spot size   

Small spot size (around 0.1–1 µm) will allow probing systems that are electronically or structurally inhomogeneous over corresponding length scales. X-ray absorption experiments using submicrometre resolution have revealed important information and we expect that the merits of RIXS will add substantial insights for better understanding the nature of inhomogenous systems. In particular, we expect that such submicrometre RIXS experiments will enable us to detect how phase transitions take place in an inhomogeneous fashion that may include nucleation phenomena.

### 8.3. Improved energy resolution   

Higher primary-beam intensity in a small spot size (0.1–1 µm) gives new opportunities for RIXS instruments facilitating much higher energy resolution. Improved energy resolution is prerequisite for a range of new RIXS experiments targeting very low energy loss of the order of a couple of 10 meV. In particular, vibrations, spin, charge and magnetic excitations will be able to be investigated in unprecedented detail. For high-temperature superconductors such energy resolution being better than the size of the superconducting gaps will make it feasible to probe the pairing symmetry and the phase of the order parameter of these superconducting materials. High-resolution RIXS maps will give a wealth of additional opportunities; for example, they provide the option to measure soft X-ray core spectra with sub-lifetime resolution, using the coherent excitation and decay. This will increase our insights into the nature of the core excited states and their various decay channels, including their interference. By using core-hole clock analysis routes this allows the determination of the time-dependent decay channels.

### 8.4. Beam damage   

Finally, we provide some remarks on the challenges to be faced with the dramatically increased X-ray dose per area at DLSR sources. Present RIXS beamlines can already create serious beam effects in sensitive materials. Molecular systems are typically destroyed during experiments within seconds or minutes and many solid systems are also affected at least on longer time scales. These beam damage effects will definitely increase with a DLSR source. However, for very high resolution experiments of the order of 10 meV resolution, for which beamline and spectrometer optics will be operated at lower efficiencies, this is expected to be less of a problem compared with high-intensity experiments. For high incident intensities it will be mandatory that DLSR RIXS experiments implement procedures to prevent/reduce beam damage. Liquid jets and other beam-based sample introduction methods are ideally suited, and for solid samples manipulators capable of rapidly moving the sample with high precision are needed.
